# Patients’ perspectives on quality and patient safety failures: lessons learned from an inquiry into transvaginal mesh in Australia

**DOI:** 10.1186/s12913-024-10791-w

**Published:** 2024-04-08

**Authors:** Mina Motamedi, Chris Degeling, Stacy M. Carter

**Affiliations:** Australian Centre for Health Engagement Evidence and Values (ACHEEV), University of Wollongong, Wollongong, NSW Australia

**Keywords:** Transvaginal mesh, Innovative surgery, Senate Inquiry, Patient harm, Quality and patient safety

## Abstract

**Background:**

Transvaginal mesh (TVM) surgeries emerged as an innovative treatment for stress urine incontinency and/or pelvic organ prolapse in 1996. Years after rapid adoption of these surgeries into practice, they are a key example of worldwide failure of healthcare quality and patient safety. The prevalence of TVM-associated harms eventually prompted action globally, including an Australian Commonwealth Government Senate Inquiry in 2017.

**Method:**

We analysed 425 submissions made by women (*n* = 417) and their advocates (*n* = 8) to the Australian Senate Inquiry, and documents from 5 public hearings, using deductive and inductive coding, categorisation and thematic analysis informed by three ‘linked dilemmas’ from healthcare quality and safety theory. We focused on women’s accounts of: a) how harms arose from TVM procedures, and b) micro, meso and macro factors that contributed to their experience. Our aim was to explain, from a patient perspective, how these harms persisted in Australian healthcare, and to identify mechanisms at micro, meso and macro levels explaining quality and safety system failure.

**Results:**

Our findings suggest three mechanisms explaining quality and safety failure: 1. Individual clinicians could ignore cases of TVM injury or define them as ‘non-preventable’; 2. Women could not go beyond their treating clinicians to participate in defining and governing quality and safety; and. 3. Health services set thresholds for concern based on proportion of cases harmed, not absolute number or severity of harms.

**Conclusion:**

We argue that privileging clinical perspectives over patient perspectives in evaluating TVM outcomes allowed micro-level actors to dismiss women’s lived experience, such that women’s accounts of harms had insufficient or no weight at meso and macro levels. Establishing system-wide expectations regarding responsiveness to patients, and communication of patient reported outcomes in evaluation of healthcare delivery, may help prevent similar failures.

**Supplementary Information:**

The online version contains supplementary material available at 10.1186/s12913-024-10791-w.

## Background

Transvaginal mesh (TVM) surgeries emerged as a new treatment for stress urine incontinency (SUI) and/or pelvic organ prolapse (POP) in 1996 [1, 2]. Details of severe adverse events emerged years after their rapid adoption into surgical practice [1–3]. Consequently, TVM-associated harms and subsequent epistemic injustice—unjust dismissal or exclusion of a person’s contribution to the production of knowledge—have become a key example of worldwide failure of healthcare quality and patient safety [1–4]. In this study we use insights from Waring and colleagues [5] to contextualise the failure to prevent harm from the use of TVM surgeries in Australia through a quality and patient safety framework.

**Fig. 1:**
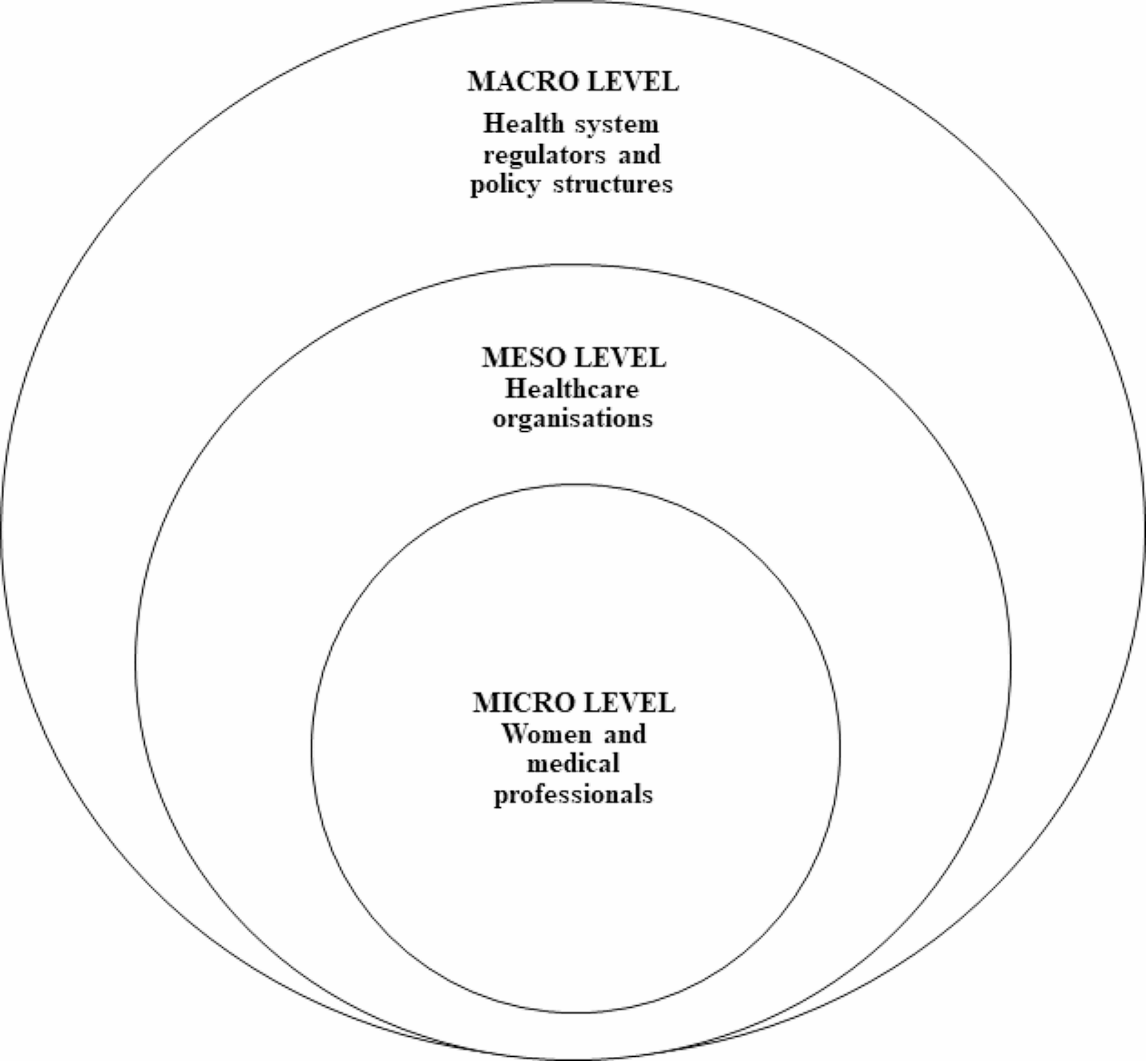
Micro, meso and macro levels of healthcare systems, drawing on Gabe et al. [9]

Quality and patient safety movements began in the 1990s, and have since gained significant traction in healthcare and politico-legal systems [5–7]. Only preventable adverse events are considered appropriate targets for quality improvement and patient safety systems [5, 8]. In their 2016 review, Waring et al. argued that healthcare quality and safety can be approached from two contrasting perspectives: an orthodox perspective or a sociological perspective [5]. Each perspective provides critical analysis of experienced preventable adverse events. But taken together, they can identify vulnerabilities in healthcare settings and instantiate learning opportunities at all three healthcare system levels—micro, meso and macro (Fig. 1) [5, 9].

The orthodox perspective recognises the interaction between human factors and local environmental factors in analysing adverse events via focusing on the micro sphere and shifting analytical attention away from individual clinicians’ failures or negligence, and towards a broader configuration of quality of care [5]. The sociological perspective provides greater consideration of the social and cultural contexts of adverse events via focusing on meso and macro insights, including social inequality and asymmetries of knowledge, and power and control [5].

While these perspectives complement one another, they also share potential points of conflict, referred to as ‘three linked dilemmas’: the epistemology of quality and safety, the culture of quality and safety, and power and politics [5]. With respect to epistemology (knowledge for quality and safety), the orthodox view assumes risks and events can be objectively and systematically measured, while the sociological view assumes that meanings of and responses to risk arise from the discourse, relationships, norms and beliefs operating in a healthcare environment. The orthodox perspective assumes that culture is something an organisation ‘has’ which can be measured and changed; the sociological perspective holds that multiple cultures emerge from the structures and interactions in complex organisations. Finally, the orthodox approach assumes healthcare can be de-politicised (e.g. through organisational strategy) whereas the sociological perspective assumes healthcare always involves complex institutionalised power differences. Waring et al. suggest that quality and safety approaches need to incorporate insights about knowledge, culture and power/politics, and that these dimensions should also be a focus of quality and safety research [5].

Our focus is on harm from the use of TVM in Australia. As in many other countries, TVM-associated adverse events in Australia eventually prompted grass-roots movements, and then an Australian Senate Inquiry in 2017 into [The] Number of women who have had TVM implants and related matters [10]. The Senate Inquiry generated copious data, mostly publicly available, including submissions from women and their advocates^1^. We analysed these data with a particular focus on women’s accounts of micro, meso and macro factors that contributed to their experience, to generate a patient-centred account of how harms arose from TVM procedures in Australia. Our analysis sought to derive and understand—from women’s perspective—the mechanisms that could explain why TVM-associated harms were neglected in the Australian health system. In developing this analysis, we relied both on data from the Senate Inquiry and insights from Waring et al. [5].

## Method

The Senate Inquiry received 555 written submissions [10]. Of these 555 submissions, only 513 submissions were made by women and their advocates. Also, 83 of these submissions were confidential, leaving 430 submissions available for analysis. Five public hearings were held in four Australian cities^2^, that women and/or their advocates spoke at these hearings. We included text of submissions, transcripts of public hearing statements, and transcripts of exchanges with senators in this analysis.

We used a case-based approach, using the individual women or advocacy organisation as the unit of analysis where possible, and combining all submissions/statements from that case into one document. Some statements during public hearings were deidentified. For this reason, each hearing transcript was included as a unique document. Within these documents, text from identified sections of these statements was linked to relevant written submissions, the remaining text was considered as one document. Our final sample was 435 documents—430 unique submissions and 5 public hearing transcripts—ranging from 1 to 263 pages in length (Table 1). An additional 26 supplementary documents and submissions, provided by women or their advocates, were combined into these 435 cases for analysis.

**Table 1:** Characteristic of included documents

Type of document		Number of documents (percentage of total included documents)		Length
Submissions made by women and/or their families	reporting TVM positive outcomes	49 (11.26%)		1 to 5 pages
	reporting TVM negative outcomes	371 (85.29%) + 18 supplementary documents		10 to 15 pages
Women	awaiting TVM procedure	2 (0.46%)		1 to 2 pages
Submissions made by patient advocates		8 (1.84%)	Submission 21	23 pages
			Submission 26	9 pages
			Submission 35	3 pages
			Submission 37	4 pages
			Submission 73	3 pages
			Submission 115 + 5 supplementary documents	118 pages
			Submission 129 + 3 supplementary documents	133 pages
			Submission 130	263 pages
Public hearings		5 public hearings (1.15%)	3 Aug 2017	58 pages
			25 Aug 2017	59 pages
			18 Sep 2017	66 pages
			19 Sep 2017	54 pages
			6 Feb 2018	19 pages

Note: Some women and their families wrote letters, others responded to a structured questionnaire created by a law firm to assist women and their advocates in responding to the inquiry. The questionnaire presented the Senate Inquiry’s terms of reference as open ended questions. This was designed to assist women to respond in a structured format [10]

Formal ethical approval was not required for this study, as all materials were in the public domain. In data collection and reporting we did not use the names of individual women and their families, relying on the numerical identifiers allocated by the Senate Inquiry process to avoid unnecessary identification. All three authors (MM, CD, and SC) contributed to the data curation. We transferred all available documents into NVivo for data management and coding. Descriptive characteristics of submissions were charted. A sample of submissions was reviewed by one author (MM), on this basis a coding matrix was developed and discussed between authors (MM, CD, and SC). The matrix combined deductive codes based on earlier analysis of the qualitative literature on women’s experience of TVM surgery, and inductive codes developed from this sample. After several iterations, a final matrix was developed by consensus between the three authors (MM, CD, and SC). MM used the final matrix to code all included documents. After completion of coding, themes were synthesised collaboratively between the three authors (MM, CD, and SC). The deductive and inductive codes were combined into categories. These were sorted to distinguish women’s accounts of micro, meso and macro factors that contributed to their experience. Our final theoretically informed thematic analysis interpreted these sets of categories in light of Waring et al.’s framework (2016). This stepwise process allowed us to develop mechanisms explaining why existing quality and safety strategies did not prevent TVM harms. All three authors—MM, CD, and SC—collaboratively contributed to interpretation and synthesis through discussion. The 21 elements of the Standards for Reporting Qualitative Research (SRQR) were used as a research checklist to report this study—see S Table in supplementary documents [11].

## Results

We first present descriptive characteristics of the submissions, then present women’s accounts of factors contributing to their experience.

### Characteristics of submissions and statements made to the Senate

Only 49 women (11.4%) reported experiencing positive outcomes following TVM procedures. These submissions echoed those of clinicians who wrote submissions advocating for TVM. They expressed alarm about possible restrictions on TVM, extolled the benefits of TVM, advocated the need for expert and skilled surgeons, and suggested TVM allowed women to live a normal life. Only two women (0.46%) who had not had the procedure made submissions. One expressed concern about safety, stating that she would wait for the Senate’s recommendations before acting; the other argued that one type of TVM procedure would suit her: that promoted by her surgeon, and previously removed from the market due to safety concerns.

Most submissions made by women and their advocates (88.14%) reported on negative outcomes from TVM. These accounts were highly consistent with reports in the existing qualitative peer reviewed literature [3]. Advocates relied heavily on women’s testimony, but also made structural arguments locating responsibility with TVM manufacturers and healthcare system governance.

### Women’s critiques of TVM procedures in Australian healthcare

Women’s critiques reinforced the epistemic injustice experienced at a micro level, and clinicians’ strong influence on the evaluation of healthcare outcomes. Based on these critiques we developed four explanations for how TVM harms were able to persist in the Australian healthcare system, which we discuss below.

#### TVM-injured women could not act beyond the micro level, and were not provided with information, help or support at this level

Recent research by Oxlad et al. and Mckinlay and Oxlad has used Senate Inquiry data to detail failures in doctor-patient communication [12, 13]. We will not repeat that analysis here, but build on it.

TVM-injured women testified that in navigating their healthcare, they felt restricted to the micro level of interacting with individual clinicians and their practice locations, and received limited information, help or support in those contexts. Women recounted relying on their treating doctors for information, and trusting them to be “the expert in this field” and serve patients’ interests. When attempting to rectify TVM-caused harms, women were not believed, were left with no options, were sometimes treated harshly by clinicians, and lost their trust in the Australian healthcare system.

*Submission 113: “Outside I was extremely upset, shaking and burst into tears. I had gone in there desperately seeking answers but I got, rejection, arrogance and a web of lies. […]I was afraid to challenge him because I understood that before discharging me he had to do an internal examination and I didn’t want angry hands to be put inside me.”*

*Submission 549: “I could no longer handle the pain, I presented by ambulance 3 times to the Hospital…. given pain medications discharged. I was told by a nurse, “We don’t treat you here, you have to go and see your Doctor for that” My blood was taken for testing and later told… good news, you don’t have cancer” I couldn’t believe what I was hearing? Same week presented by ambulance to the Hospital with a referral from my GP. I had to refuse to go home until I had a colonoscopy. The treatment or lack of, I received at Hospital left a lot to be desired. Discharged with no answer only drug relief and the side effects nearly killed me.” […] I got my Hospital records and found, Doctor […] had implanted me with […] Mesh plus a TVT-O […] Without My Knowledge or Consent. Doctor […] referred me to other Specialists hoping to enlist their help with operating on me. One Doctor report said, I needed [psychological] help, another said, I needed to learn pain management, another said, he’d just cut the arms to the Bladder sling. […] Another Specialist I went to, shouted at me as I was hobbling from his room in shock from his brutal examination saying, he never wanted to see me again.”*

When the Senate Inquiry asked “how affected women can tell their stories to their doctors?”, many women responded in sarcastic language, noting, for example, that this is the *“million-dollar question”*^3^, that their concerns would be dismissed if expressed, and their reported symptoms would be dismissed as being “in their heads”, rare and unfortunate, or normal post-operative experience.

*Submission 110: “Women being told that they are the only ones to have had any adverse effects, and/or it is all in their heads, and being treated with disdain. This has caused many women to just suffer through their problems and give up on seeking help.”*

*Submission 280: “Dr. dismissed my concerns, and advised me that I would ‘never have a normal bladder’ again after this procedure and that the side effects I was experiencing were normal.”*

*Submission 538: “It’s all in my head they said, You have to live with it. There’s not much we can do My self-worth has gone!”*

Also at the micro level, women reported clinical restrictions when removal surgery was considered high-risk or impossible, and issues with options not being explained clearly or clinicians telling untruths about what procedures had occurred. Women’s accounts of loss of trust drew on both the severity of harms and delayed or inappropriate responses to harms.

*Submission 498: “The mesh I have can’t be fully removed safely here in Australia as it’s too dangerous and I have been told this by two different specialists. They have said “It’s like cutting reo out of concrete without damaging the concrete.”*

*Submission 321: “to date no-one has enough experience in Australia for me to put my trust in them. I’m so scared I am going to die.”*

*Submission 147: “When I finally obtained my operation report from the hospital, […], I discovered that my Surgeon had not actually performed the operation as he had promised […]. His Registrar performed the operation […] I also discovered […] that I had been implanted with […] the mesh that had already been in the spotlight […] for causing injuries when implanted in women. It was now clear he had blatantly lied to me. I made a conscious decision never to go back to my Surgeon and found myself in the depths of depression and despair not knowing who to trust in the medical profession.”*

*Public hearing 3 August 2017, page 6: “the only thing that I know is available […] is a very old procedure called the Burch colposuspension […]. It is quite invasive. I have quite substantial pelvic scarring and I have actually been informed by my removing surgeon that even that or a facia or an own tissue repair that they could provide would probably fail as well, given the extreme damage to my pelvic floor.”*

The Senate Inquiry asked about public reporting to the Therapeutic Goods Administration (TGA) or other healthcare system authorities. Women responded that they were unaware of or unable to use these channels. Even women who knew they could report to the TGA (e.g. because they were healthcare workers, or were notified via media or TVM support groups) said the process was too complicated and/or required detailed information about the specific device used—which women could not obtain. Women asserted that the self-regulatory, non-mandatory system for health services reporting adverse events encouraged providers to put profits before patient safety, allowing under-reporting or delay reporting harms. Thus, institutions and clinicians could prioritise their own interest over patients’ interests. Women highlighted other meso and macro restrictions on redress, including long waiting lists and procedure costs limiting access to TVM removal.

*Submission 227: “…was sent to hospital and still waiting to hear from doctor at […] hospital that said it wasn’t urgent (sorry don’t agree) still waiting after approx. 5 years”*.

*Submission 257: “The cost of this procedures would be well beyond our means it has been quoted at around 50,000 dollars and on one income would be a financial burden on our already stretched finances”*.

Women expressed gratitude that the Senate Inquiry provided an opportunity for macro-level agency and validation of their experience. They requested urgent financial and non-financial help and support, including: TVM multidisciplinary clinics; a database of TVM specialists and their outcomes to support informed decision-making; and accessible, effective and free TVM-removal surgeries. Women who had experienced total TVM-removal surgery, even if they had required multiple surgeries or had not had complete resolution of symptoms, advocated for total removal as early as possible.

#### Some clinicians’ performance was inconsistent with quality and safety standards, but this was not detected

Most women acknowledged that evidence of potential adverse events was emerging at the time of their operations. However, as reported by Oxlad et al., most women engaged with the Senate Inquiry to report conduct that was inconsistent with accepted practice standards [12]—and would be so even in the context of providing safe and effective treatment. Oxlad et al.’s analysis [12] supports our second proposed explanation for the persistence of harm: that clinicians’ practices were inconsistent with quality and safety standards, but this was not detected. In addition to promotion of harmful procedures, women reported micro level failures including failure to obtain valid consent (including failure to explain and obtain consent for the implants used), failure to follow up, failure to document and investigate reported symptoms, and blaming, gaslighting or acting in offensive ways to women who raised concerns.

#### Thresholds to trigger reporting and investigating harms were inappropriate

The third explanation for the persistence of TVM harm concerns the thresholds in organisations (meso) and regulatory organisations (macro) that triggered reporting and investigation of harm. Women suggested that in other cases of harmful treatments, small absolute numbers had triggered investigation and change. Thalidomide was a common example, which was withdrawn from the market based on reports of about 200 adverse events. Women provided data collated by support groups to suggest that there were more than 2,400 TVM-injured women in Australia, so by analogy, action should have come much sooner.

*Public hearing 3 August 2017, Page 18: “if we go back to the previous frontrunner in catastrophic outcomes in the health system, thalidomide, there were only—and when I say ‘only’, this is by comparison—about 200 infants born with deformities in Australia, and that is all it took to take it off the market immediately. […] The very absolute numbers […] 2,400 women whose lives have been ruined. That is 10 times as many whose lives were ruined by thalidomide. Why is it that we now still believe that that is a reasonable outcome, that they are reasonable odds?”*

Women often asserted that *“one woman harmed by mesh is one too many”*^4^, highlighting the effect on not just women but their families, and proposing a total ban on use of TVM procedures to prevent further harm. It was also argued that thresholds and evaluation focused on short-term outcomes and ignored long-term outcomes, and over-relied on reports by treating clinicians.

*Submission 21: “From the surgeon’s perspective, surgery is less invasive, day stays are shorter and the holding up of organs (seen as the goal of the surgical procedures […]) is achieved. The potential for long-term side effects are largely ignored, or deemed by the surgical community to affect an insignificant number of women.”*

#### System design issues worsened TVM-associated harms

The final explanation for the persistence of TVM harms concerns system design issues which stripped women of agency and autonomy, in part by exacerbating the harms they had already experienced. These included extensive waiting times for appointments, insufficient allocated time for individual healthcare delivery, ineffective communication within multidisciplinary teams and lack of transparency, all of which limited women’s opportunities to escalate their concerns.

*Submission 185: “a 15-minute consultation is not even close to long enough.”*

Poor medical records and lack of access for women to their own medical records contributed to ineffective communication and interfered with women’s options to take legal action.

*Submission 423: “I visited two Specialists regarding the vaginal Bleeding and they could not put any light on it as my Medical Records which they were given did not have any details of the previous surgeries on it. I then rang the DR who inserted mesh but he would not divulge anything, I then rang the Dr who carried out the 3rd repair who then told me that I definitely had the mesh there and she tried to trim what she could. Then I enquired at my Drs surgery and asked to see the reports they had but had not been put into my records.”*

*Submission 525: “I rang my gynaecologist from 2002 & asked him if he could remember what tape he used on me in 2002 or if he had written evidence but he said he thinks it was. had no evidence. So I had nowhere to go with that one even though I had years of suffering.”*

When complaints were made against clinicians, these were settled in non-transparent ways via the complaints system, and were between the woman and an insurer rather than the woman and the treating clinician.

*Submission 163: “…Complaints were made to the HCCC re my matter but it went nowhere. So after almost 3 − 1/2 years later, I will be attending an informal Settlement Conference—not with the doctor in question, but with… insurance company. How wrong is this? In all probability a monetary settlement will be reached but the public will never know what happened to me as it will all be swept under the carpet and then what happens to the next patient, who is not aware of this doctor’s prior history?”*

Women requested changes to system design, including a stronger regulation and audit system, greater support and education for clinicians, a national registry of medical devices, and clear direction regarding accountabilities and responsibilities for device-caused harms to mitigate these failures across three levels.

## Limitations

The availability of public domain data from the Senate Inquiry provided a unique opportunity to develop systematic insights into this episode of failure in healthcare quality and safety without further burdening the people who had already been harmed. However, relying on these data also introduces limitations. We were unable to reflect demographic or other characteristics of women in our analysis. We note also that the Senate assumed all users of gynaecology services were women, without asking them to self-identify. We were also limited by the structure of the inquiry itself—we were able to use only data from those women who were able to participate in the Senate Inquiry, and the form of these data was constrained by the standardised data collection process. It is likely that the most marginalised women affected by TVM-associated harms had greater difficulty participating in the Senate Inquiry, and so will not be included in this analysis. There was also greater engagement with the Inquiry from harmed women—we know that many other women experienced benefit from TVM procedures. Our findings should be interpreted with these limitations in mind.

## Discussion

**Fig. 2:**
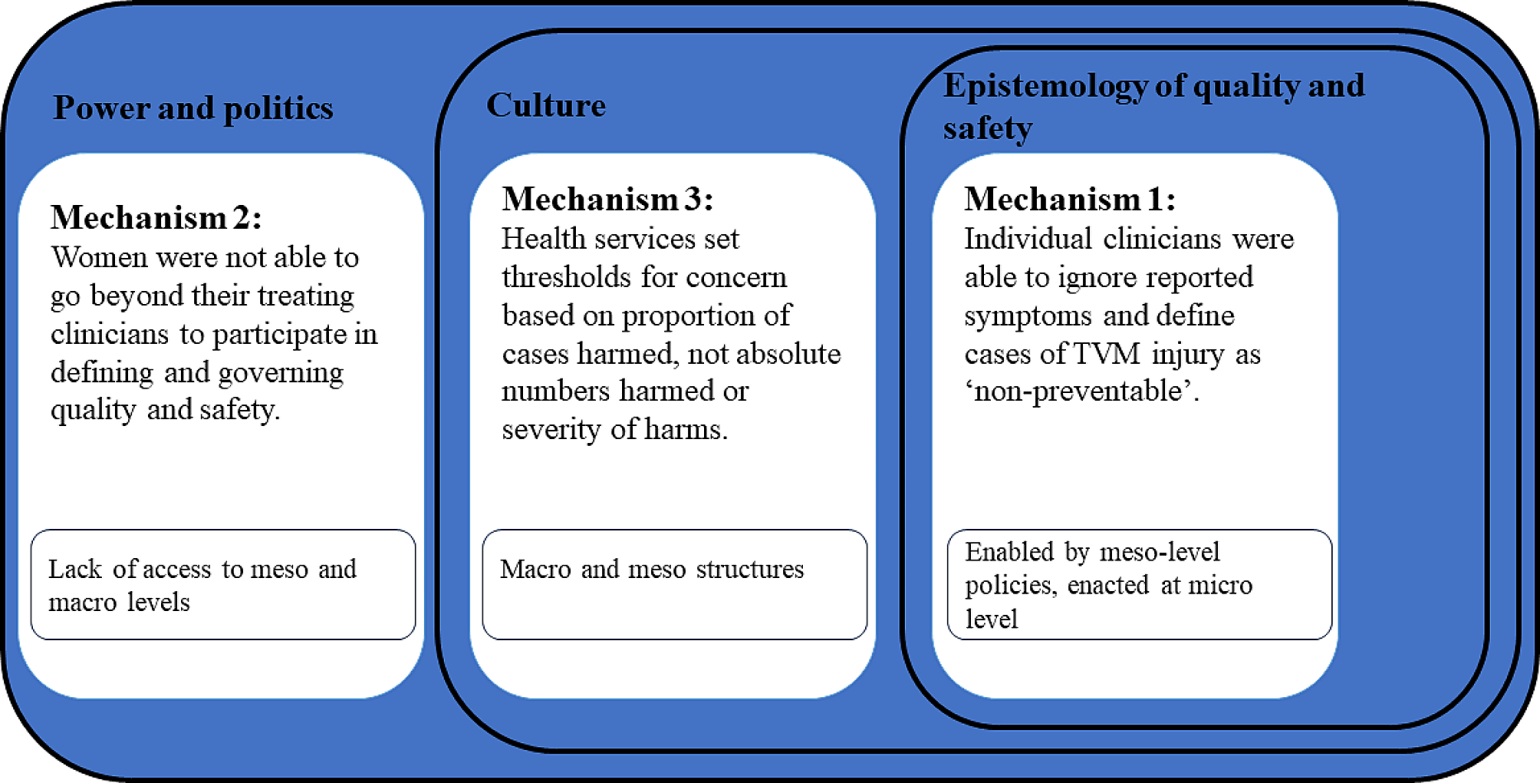
Mechanisms explaining healthcare quality and safety failures in TVM procedures in Australia, drawing on Waring et al. [5] and the accounts of women and their advocates

In our findings, we provided four explanations, on women’s account, for how TVM-associated harm was able to persist in the Australian healthcare system. We now return to Waring et. al.’s dilemmas— epistemology of quality and safety, culture, and power and politics—in the context of quality and safety [5]. We used this conceptual framework to draw together elements of the explanations presented in our findings to propose three mechanisms that help to explain failure to respond to TVM-associated severe, low-incidence harms in the Australian healthcare system (Fig. 2):

Mechanism 1: Individual clinicians were able to ignore reported symptoms and define cases of TVM injury as ‘non-preventable’.

Mechanism 2: Women were not able to go beyond their treating clinicians to participate in defining and governing quality and safety.

Mechanism 3: Health services set thresholds for concern based on proportion of cases harmed, not absolute numbers harmed or severity of harms.

These mechanisms were developed to capture what the case of TVM can offer quality and safety science beyond well-acknowledged challenges (e.g. the need for informed consent, and for effective and respectful communication between clinicians and patients). We will discuss the three mechanisms and link them to Waring et. al.’s dilemmas [5] in turn, showing how they can support action to improve quality and safety systems. As shown in Fig. 2, we argue that each mechanism is providing evidence of implications for one identified dilemma by Waring et al. [5].—epistemology, culture, and power/politics of quality and safety.

### Mechanism 1: individual clinicians were able to ignore reported symptoms and define cases of TVM injury as ‘non-preventable’

On women’s accounts, individual treating professionals were able to determine whether reported symptoms were real and a case of TVM-related harm, and thus whether an event should be reported as a quality and safety incident or counted as ‘non-preventable’ and not a reportable incident. This included clinicians being able to choose not to record adverse outcomes at all, whether they surfaced in hospital, in post-discharge follow up, or later. This mechanism arose from the privileged position of clinicians, afforded by health service structures. Meso-level policies and processes enabled clinicians to delay or block responses to women’s reported harms at the micro level, including not escalating reports of harms to safety specialists.

We argue that this Mechanism is relevant to epistemology of quality and safety. The micro level failure of not recognising TVM-associated harms as a patient safety issue hindered escalation of TVM-injured women’s testimonies to meso level. This meant safety specialists were not notified, so cause-and-effect analyses were not conducted, and quality and safety procedures were not initiated within individual organisations. On women’s accounts, this was a significant epistemic barrier to governing these innovative procedures. Once TVM-associated harms were recognised as significant, this changed: evidence of harm was collected, communicated, stored, and used at meso and macro levels to adjust clinical guidelines.

Ducey and colleagues suggest that variation in clinicians’ performance, perceptions and experiences have direct impacts on what counts as a preventable or non-preventable adverse event [14]. This, in case of TVM, particularly highlights a weakness of quality and patient safety—only targeting preventable adverse events. Here, some clinicians chose not to report these events at all, and this autonomy and agency to shape what counts at an individual level was, on women’s accounts, a substantial barrier to effective governance. Building on Waring et. al.’s argument, Bosk and Pedersen noted the gap that can exist between safety specialists’ perceptions of adverse events and patients’ actual experience of adverse events, in part because of measures and definitions, reinforces the risks of relying solely on clinicians’ evaluations, and the need for patient reported outcome measures and patient involvement in evaluation [5, 15]. An equivalent inquiry in the UK in 2020 acknowledged dismissal of patient’s testimony as a primary theme, and recommended the appointment of a Patient Safety Commissioner to provide an independent channel to listen to patients [16]. We acknowledge that the Australian healthcare system is moving towards greater emphasis on the importance of communicating all adverse events, to ensure continuous quality improvement and patient safety in healthcare delivery. However, this may have not been the case during the rapid introduction and adaptation of TVM procedures into practice.

For example, Clinical Excellence Commission in New South Wales has identified adverse events (including serious morbidity) as one of key trigger and criteria for development of a systematic agenda for Morbidity and Mortality Review Meetings, and informing clinical analytics [17]. Also, at national level, the Australian Commission on Safety and Quality in Health Care (ACSQHC)^5^ has introduced strategies including an Accreditation Scheme, National Safety and Quality Health Service (NSQHS) Standards, and Patient safety surveillance programs [18]. This includes development of specified patient safety measures and indicators for sentinel events, adverse events, core hospital-based outcome indicators, etc. Sentinel events are defined as rare and wholly preventable events that result in serious harm to, or death of, a patient [18]. In Australia reporting of sentinel events, has been mandatory since 2007. However, screening and reporting of other/non-preventable adverse events is voluntary and not mandatory [18]. ACSQHC has initiated development of a national online audit and surveillance platform (commencing 2019) to improve screening for all adverse events [18]. This movement towards greater audit and surveillance is consistent with our broader recommendation: that strengthening quality and patient safety should not rely entirely on the judgement of clinicians about the preventability of adverse events.

### Mechanism 2: women were not able to go beyond their treating clinicians to participate in defining and governing quality and safety

This mechanism is linked to our finding that women were not only restricted and stripped of agency at a micro level. They also had limited access to meso and macro levels: women were not aware of, or able to engage with, agencies or authorities beyond their clinicians. This meant women’s perspectives could not inform decision making at the meso or macro levels to influence definitions of what should be counted as TVM-associated harms.

While Mechanism 1 was strongly focused on the epistemology of quality and safety, Mechanism 2 was especially a product of power and politics in quality and safety. Mechanism 2 demonstrates strong reliance on clinicians’ evaluations, and restriction of women’s communication only to the micro level. This hampered changes to clinical governance or guidelines that could have altered clinical practice at a micro level or changed shared understandings of TVM procedures. Critiques of quality and safety have highlighted the power of clinicians to influence what counts as a quality and patient safety issue within all levels of healthcare settings [6, 14, 15, 19]. This also resonates with the well-recognised history of power imbalance in the construction of knowledge of the female body in medicine and surgery [19]. Waring et al. argued that power differences will always exist in healthcare systems [5]. In this case, it is clear that TVM-injured women had no power or less power than clinicians, which meant that they were less able to access meso and macro levels of the healthcare system to effect change in understandings of their condition. We argue that power/politics relates to Mechanisms 1–3. This included defining cases out of the quality and safety system by classifying reported harms as imaginary, normal post-operation experience, or ‘non-preventable’ and not a reportable incident. In the UK review, this need for substantial revisions at meso and macro level to ensure effective engagement of patients and communication of their reported outcomes were also highlighted, including the need to revise regulatory systems to support greater patient engagement [16]. 

section3

**Mechanism 3: Health services set thresholds for concern based on proportion of cases harmed, not absolute numbers harmed or severity of harms**.

Mechanism 3 both arises from and reinforces Mechanisms 1 and 2. Interacting with the epistemic problem of defining harm, and the political problem of having access to participate in systems for defining and governing quality and safety, was a cultural problem. As Waring et al. notes, culture is not a static thing that an organisation ‘has’—rather, it is a dynamic and co-created feature of any healthcare system or unit [5].

On women’s accounts, this Mechanism was a system issue operating primarily at meso and macro levels. Policy contexts allowed individual healthcare services to set thresholds for concern that focused on the proportion of cases harmed. Women argued that this allowed dismissal of the severity of experienced harm or absolute number of cases harmed and variable responses to reported adverse events, insufficient collation of data, and inappropriate and delayed responses. We suggest this Mechanism corresponds to Waring et al.’s dilemma of culture [5]. In the case of TVM harms, individual work unit cultures allowed instances of severe harm to go unreported and unmitigated. It was only after many years of collective action to raise awareness [20] that TVM-injured women were able to request an appropriate response to the harms they had experienced. On women’s account, this was a failure for the women themselves, but also at the meso and macro levels of the healthcare system. The self-regulatory, non-mandatory system for health services reporting adverse events encouraged individual healthcare services’ culture to use different threshold for concern.

While acknowledging and agreeing with Waring et. al.’s observations about culture [5], we argue that in this instance a culture of safety and reporting does require nurturing and leadership at micro, meso and macro levels. Without establishment of some system-wide expectations regarding communication, appropriate evaluation and assessment of healthcare delivery, and responsiveness to patients, there is likely to be inequity in the way healthcare organisations respond to harm. We note that this is unlikely to be limited to TVM, as demonstrated by evidence of harms associated with other innovative surgical implants emerging years after their adaptation into practice, such as metal-on-metal hip implants and poly implant prosthese breast implants [21]. Higher expectations of system accountability, should arguably be stronger in areas of practice with high possibility of causing iatrogenic harms, including in innovative surgical procedures that carry risk of unknown possible adverse events.

## Conclusion

The prevalence of TVM-associated harms remained unknown for almost two decades in Australia. We derived three mechanisms to help explain failure of the system to appropriately respond to these harms. Epistemically, individual clinicians were able to define whether a harm was a harm. Politically, women could not participate in the systems that defined and evaluated harm. And culturally, work units and organisations were able to dismiss reported issues and not communicate them to meso and macro levels.

From our analysis it appears that clinical perspectives were privileged over patient perspectives in evaluation of healthcare outcomes in this case, and that the meso and macro levels of the health system were unknown to women. This case demonstrates the need for several health system interventions. Public and patient involvement should continue to be strengthened at all levels of healthcare systems to enable direct communication of patient-experienced outcomes. Care should be taken regarding who has the epistemic authority to recognise iatrogenic harms or pronounce them non-preventable. Communication of all adverse events reported by patients at all levels of healthcare systems—particularly as data-intensive techniques improve—should allow faster collation, analysis and identification of severe harms. Finally, greater attention to how thresholds for action are set within those systems should enable faster and more responsive action. Lessons learned from women’s accounts of TVM-associated harms in Australia highlight the need for establishment of expectations for responsiveness to patients and communication of their reported outcomes throughout the healthcare system. This would enhance evaluation and assessment of healthcare delivery, particularly in use of innovative surgical procedures that carry risk of unknown possible adverse events.

### Electronic supplementary material

Below is the link to the electronic supplementary material.

Supplementary Material 1

## Data Availability

All data and used materials for this study were in the public domain. Provided by the Australian Parliament Senate Community Affairs References Committee: https://www.aph.gov.au/Parliamentary_Business/Committees/Senate/Community_Affairs/MeshImplants/Report. In data collection and reporting we did not use the names of individual women and their families, relying on the numerical identifiers allocated by the Senate Inquiry process to avoid unnecessary identification.

## Footnotes

1 Patient advocates group includes Health Consumers Councils across Australia (HCC), Continence Foundation of Australia (CFA), Consumer Health Forum of Australia (CHF), Scottish Mesh Survivors Group (SMSG), Mesh Down Under (from NZ), Health Issues Centre (HIC), Australian Pelvic Mesh Support Group (APMSG), Sling The mesh campaign (from UK).

2 Five public hearings were held in Melbourne, Perth, Sydney, and Canberra.

3 Submission 221, page 4.

4 Public hearing 19 September 2017, page 36.

5 the Australian Commission on Safety and Quality in Health Care (ACSQHC) was established, in 2006, by the Council of Australian Governments (COAG) to lead and coordinate national improvements in the safety and quality of health care. The Commission works in partnership with patients, carers, clinicians, the Australian, state and territory health systems, the private sector, managers and healthcare organisations to achieve a safe, high-quality and sustainable health system.
