# Supplementary material for: Patients’ perspectives on quality and patient safety failures: lessons learned from an inquiry into transvaginal mesh in Australia
Source: BMC Health Serv Res. 2024 Apr 8;24:436. doi: 10.1186/s12913-024-10791-w (PMC11005218; doi:10.1186/s12913-024-10791-w)
Supplement: Supplementary file 1 — Supplementary Material 1 [file 12913_2024_10791_MOESM1_ESM.docx]

# Supplement - Research checklist

We used the 21 elements for the Standards for Reporting Qualitative Research (SRQR)^[[1]](#footnote-1)^ to report our study- see S Table.

S Table - An Adaptation of the 21 Elements of O’Brien and Colleagues’ Standards for Reporting Qualitative Research (SRQR)

| Standard 1 |  | Title |
| --- | --- | --- |
| Standard 2 |  | Abstract |
| Standard 3 |  | Problem Formulation |
| Standard 4 |  | Purpose or research question |
| Standard 5 |  | Qualitative approach and research paradigm |
| Standard 6 |  | Researcher characteristics and reflexivity |
| Standard 7 |  | Context |
| Standard 8 |  | Sampling strategy |
| Standard 9 |  | Ethical issues pertaining to human subjects |
| Standard 10 |  | Data collection methods |
| Standard 11 |  | Data collection instruments and technologies |
| Standard 12 |  | Units of study |
| Standard 13 |  | Data processing |
| Standard 14 |  | Data analysis |
| Standard 15 |  | Techniques to enhance trustworthiness |
| Standard 16 |  | Synthesis and interpretation |
| Standard 17 |  | Links to empirical data |
| Standard 18 |  | Integration with prior work, implications, transferability, and contribution(s) to the field |
| Standard 19 |  | Limitations |
| Standard 20 |  | Conflicts of interest |
| Standard 21 |  | Funding |

1. O’Brien BC, Harris IB, Beckman TJ, Reed DA, Cook DA. Standards for reporting qualitative research: a synthesis of recommendations. Academic medicine. 2014 Sep 1;89(9):1245-51. DOI: 10.1097/ACM.0000000000000388 [↑](#footnote-ref-1)
